# WHO releases global COVID-19 vaccination strategy update to reach unprotected

**Published:** 2022-08

**22 July 2022** - WHO published an update to the Global COVID-19 Vaccination Strategy today, in response to the spread of Omicron subvariants, advances in vaccine evidence, and lessons from the global vaccination program.

In the first year of rollouts, COVID-19 vaccines are estimated to have saved 19.8 million lives. Through unprecedently large and rapid rollouts worldwide, over 12 billion doses have been administered globally, in nearly every country in the world, resulting in countries reaching 60% of their populations on average.

Yet only 28% of older populations and 37% of health care workers in low-income countries have been vaccinated with their primary series. 27 of WHO’s Member States have not yet started a booster or additional dose program, 11 of which are low-income countries.

The strategy aims to use primary and booster doses to reduce deaths and severe disease, in order to protect health systems, societies and economies. On the way to reaching the 70% vaccination target, countries should prioritize achieving the underpinning targets of vaccinating 100% of health care workers and 100% of the most vulnerable groups, including older populations (over 60s) and those who are immunocompromised or have underlying conditions.

“Even where 70% vaccination coverage is achieved, if significant numbers of health workers, older people and other at-risk groups remain unvaccinated, deaths will continue, health systems will remain under pressure and the global recovery will be at risk,” said WHO Director-General Dr Tedros Adhanom Ghebreyesus. “Vaccinating all those most at risk is the single best way to save lives, protect health systems and keep societies and economies open.”

To ensure vaccines reach the highest priority groups, the strategy emphasizes the need for measuring progress in vaccinating these groups and developing targeted approaches to reach them. Approaches include using local data and engaging communities to sustain demand for vaccines, building systems for vaccinating adults, and reaching more displaced people through humanitarian response.

The strategy also has the goal of accelerating development and ensuring equitable access to improved vaccines to substantially reduce transmission as the top priority but also to achieve durable, broadly protective immunity.

Current vaccines were designed to prevent serious illness and death, which they have succeeded in doing, saving millions of lives. However, they have not substantially reduced transmission. As the virus continues to circulate widely, new and dangerous variants are emerging, including some which reduce the efficacy of vaccines. It is fundamental to continue investing in research and development to make more effective, easier to administer vaccines, such as nasal spray products.

Other vital actions to take include: equitably distributing manufacturing facilities across regions and supporting strong vaccine delivery programs. WHO will continue to collaborate with COVAX and COVID-19 Vaccine Delivery Partnership (CoVDP) partners to support countries with rollouts, such as through packaging COVID-19 vaccination with other health interventions.

Available from: https://www.who.int/news/item/22-07-2022-who-releases-global-covid-19-vaccination-strategy-update-to-reach-unprotected

